# 

**Published:** 1887-06

**Authors:** 


### BOOK NOTICES.

TRIFET'S MONTHLY GALLAXY OF MUSIC, is a remarkably cheap and well-ordered publication. Price $1.00 per annum, single copies 10 cents. The April number contains eight vocal and eight instrumental pieces; among the former "Can You, Sweet Heart Keep a Secret?" Answer by letter inclosing $1 for a year's subscription to F. Trift, Publisher, 408 Washington Street, Boston.

For sale by all news dealers.

THE YOUTH'S COMPANION.—Since the days of "Peter *Parley*," no serial has been published so instructive and amusing to young people as the above. We never take it up without reflecting that such a weekly visitor would have been a blessing to our youth. A late number contains a very interesting piece of auto-biography by the well-known author Wm. B. Howels, beautifully illustrated, worth more than a year's subscription. Published by Perry Mason & Co., Boston Mass.

DRESS.—The above is the title of a new monthly conducted by Annie Jenness Miller, which has just made its courtesy to the public. It is "devoted to the practical and beautiful in women's and children's clothing ; Physical culture and kindred subjects," and we doubt not these objects will be faithfully served, as Mrs. Miller is not only a lady of high intellectual attainments, but an enthusiastic advocate of dress reform, who has put her theories into practice and already given them great popularity among our health-seeking classes, who are not called upon to make any sacrifice, even in fashion or style, by their adoption. The initial number is amply illustrated, and this is to be a feature of succeeding numbers.

Published by the Gallison and Hobron Company, New York.

HEALTHY HOMES AND FOODS FOR THE WORKING CLASSES.—We have received from the American Public Health Association, a neatly bound volume bearing the above title, and containing the four Essays, known as the Lomb Prize Essays, provided for by the generosity of Mr. Henry Lomb, of Rochester N. Y, which treat of healthy homes, foods, sanitary measures &c., for the working classes ; a most excellent and timely collection of rules and regulations for the preservation of health by rational and inexpensive means, which will be found of value to all classes. Price of bound volume $1.00—unbound, 60c.—Single Essays 15c. Address,

DR. IRVING A. WATSON M. D,,
Secretary, Concord N. H.

